# Impact of seasonal variation, age and smoking status on human semen parameters: The Massachusetts General Hospital experience

**DOI:** 10.1186/1743-1050-1-2

**Published:** 2004-09-30

**Authors:** Zuying Chen, Linda Godfrey-Bailey, Isaac Schiff, Russ Hauser

**Affiliations:** 1 Vincent Memorial Obstetrics & Gynecology Service, Andrology Laboratory and In Vitro Fertilization Unit, Massachusetts General Hospital, Boston, Massachusetts USA; 2 Department of Environmental Health, Occupational Health Program, Harvard School of Public Health, Boston, Massachusetts USA

## Abstract

**Background:**

To investigate the relationship of human semen parameters with season, age and smoking status.

**Methods:**

The present study used data from subjects recruited into an ongoing cross-sectional study on the relationship between environmental agents and semen characteristics. Our population consisted of 306 patients who presented to the Vincent Memorial Andrology Laboratory of Massachusetts General Hospital for semen evaluation. Sperm concentration and motility were measured with computer aided sperm analysis (CASA). Sperm morphology was scored using Tygerberg Kruger strict criteria. Regression analyses were used to investigate the relationships between semen parameters and season, age and smoking status, adjusting for abstinence interval.

**Results:**

Sperm concentration in the spring was significantly higher than in winter, fall and summer (*p *< 0.05). There was suggestive evidence of higher sperm motility and percent of sperm with normal morphology in the spring than in the other seasons. There were no statistically significant relationships between semen parameters and smoking status, though current smokers tended to have lower sperm concentration. We also did not find a statistically significant relationship between age and semen parameters.

**Conclusions:**

We found seasonal variations in sperm concentration and suggestive evidence of seasonal variation in sperm motility and percent sperm with normal morphology. Although smoking status was not a significant predictor of semen parameters, this may have been due to the small number of current smokers in the study.

## Background

Male factor infertility has been identified as the main or secondary cause in >40% of infertile couples. The number of office visits each year for infertility in the United States has risen from 600,000 in 1968 to 2 million by the 1990's. Semen analysis is a central component in male infertility evaluation [1,2]. The present study was designed to evaluate the relationship of human semen parameters to season, age and smoking status.

Seasonal variations in semen parameters have been reported in both fertile and infertile men [3-6]. Saint Pol *et al. *found a significant seasonal variation in sperm count, with highest sperm counts in late winter and early spring and lowest concentration in late summer [5]. There are several studies that suggest that an increase in age is associated with a decline in semen parameters [6-8]. Paulson and coworkers identified an inverse association between age and total sperm count, but did not find an age related decrease in fertilization rate or a decrease in live birth rate in the oocyte donation model [9].

There is a consistent association between cigarette smoking and fertility in women [10-13]. However, the data on cigarette smoking and measures of male fertility are less clear. Künzle *et al. *found an association between smoking and reduced semen quality [14] while others found no strong relationship [15].

The present study explores the relationship of human semen parameters with season, age and smoking status, while controlling for abstinence interval. In an earlier publication, we reported on the relationship between semen parameters, season, and age using data from a retrospective review of an existing laboratory database at the Massachusetts General Hospital Vincent Memorial Andrology Laboratory [16]. The present study uses data from subjects recruited into an ongoing study exploring the relationship between environmental agents and semen parameters. There is no overlap with subjects from our earlier study.

## Methods

### Study participants

The Human Subject Internal Review Board committees of the Harvard School of Public Health and the Massachusetts General Hospital approved the study (IRB #96-7545). Informed consent was obtained from each participant before entering the study.

The study population consisted of patients (n = 306) presenting to the Vincent Memorial Andrology Laboratory of Massachusetts General Hospital (MGH) between January 2000 and January 2002 for semen analysis as a component of infertility evaluation. Of the 306 subjects, age was not available for five study subjects despite several attempts at follow up after the initial study visit. Sperm morphology was not performed on two subjects with azoospermia.

Men were approached only if the referring physician had previously received a brief description of the study. The Andrologist approached patients and asked if they were willing to meet the research nurse to learn about the research study, noting that it was optional and not part of their clinic appointment. All patients approached had the opportunity to decide for themselves if they wanted to participate in the research study. Inclusion criteria for this study were: 1) scheduled for a routine semen analysis as a patient at the Vincent Memorial Andrology Laboratory, 2) either English or Spanish speaking, 3) age 18–54 years, 4) had not had a vasectomy, and 5) was not currently receiving hormone therapy.

All subjects received an explanation of the study in a private setting prior to obtaining informed consent. Information on lifestyle, such as smoking history and medical history was obtained by nurse interview.

From the date that semen specimen was produced, samples were classified by season as follows: winter = December, January, February; Spring = March, April, May; Summer = June, July, August; and Fall = September, October, November.

### Collection of samples

Semen was collected by masturbation in a private room in the hospital. The semen specimen was allowed to liquefy for at least 20 minutes in an incubator at 37°C and was analyzed within 60 minutes after collection. A routine semen analysis was performed which included the following parameters: semen volume, pH, sperm concentration, sperm motility, and sperm morphology.

### Laboratory evaluation

#### Semen volume and pH

Volume was determined and sample color and viscosity were recorded. Semen pH was measured within one hour of ejaculation (Color pHast; EM Science, Gibbstown, NJ, USA).

#### Concentration and motility

All fresh semen samples were analyzed for sperm concentration and motion parameters by the HTM-IVOS Semen analyzer (Hamilton-Thorn 10HTM-IVOS, Beverly, MA, USA). Setting parameters and the definition of measured sperm motion parameters for CASA were established by the manufacturer: (frames acquired: 30; frame rate: 60 Hz; straightness (STR) threshold: 80.0%; medium VAP cutoff: 25.0 um/s; and duration of tracking time: 0.38 sec). To measure both sperm concentration and motility, aliquots of semen samples (5 μl) were placed into a pre-warmed (37°C) Makler counting chamber (Sefi – Medical Instruments, Haifa, Israel). A minimum of 200 sperm from at least four different fields was analyzed from each specimen. Percent motile sperm was defined as World Health Organization (WHO) grade "a" sperm (rapidly progressive with velocity ≥ 25 μm/s at 37°C) plus "b" grade sperm (slow/sluggish progressive with velocity ≥ 5 μm/s but < 25 μm/s) [17].

#### Morphology

Using the "feathering" method at least two slides were derived from each fresh semen sample [17]. The resulting thin-smear slide was allowed to air dry before staining which was carried out using a Diff-Quik staining kit, consisting of three solutions (Dade Behring AG, Düdingen, Switzerland). Slides were then mounted with a microscopy cover glass (Fisher Scientific, Pittsburgh, PA, USA).

Morphological assessment was performed at a magnification of 100X with an oil immersion using a Nikon microscope (Nikon Company, Tokyo, Japan). As the slide was scored, the normal and abnormal spermatozoa (head defects, midpiece defects and tail defects) were noted. Sperm morphology was determined using the strict criteria by Kruger *et al. *[18]. From each sample at least 200 spermatozoa was counted from two slides. Results were expressed as percentage of normal spermatozoa, head defects, midpiece defects and tail defects.

### Statistical analysis

Multiple regression analyses (SAS version 8.2, SAS Institute, Cary, NC, USA) was performed to investigate whether there were differences in semen parameters with respect to season, age, and smoking status. Winter was used as the reference season. We also investigated month-to-month variation in semen parameters. For each semen parameter, a separate multiple regression was performed. Semen analysis parameters were entered into the models both untransformed and after square root transformation because of their skewed distribution. Since the square root transformed results were similar to the untransformed results, and are simpler to interpret, only the untransformed results are presented. To explore whether the semen parameters and age relationships were linear, age was used as both a continuous and categorical variable (less than 30 years, 30 to 40 years, and greater than 40 years old). Abstinence time was modeled as an ordinal five-category variable (2 or fewer days, 3, 4, 5, and 6 or more days).

## Results

Subject's ages ranged from 18.2 to 54.3 years. The mean (± SD) age was 35.9 (± 5.6) years. The majority of the subjects (67%) were between 30 and 40 years old; only 11% were <30 years and 21% were >40 years old.

Mean sperm concentration was above the WHO reference values [17]. Average (± SD) sperm concentration was 103 (± 95.5) M/ml with range = 0 – 655.4 M/ml. Mean (± SD) percent motility and percent normal morphology were 47.6% (± 25.5) and 6.9% (± 4.7), respectively. Mean (± SD) semen volume was 3.0 ml (± 1.6), with a range = 0.1 – 11.0. The mean (± SD) semen pH was 8.4 (± 0.3), with range = 6.8 – 9.0. Mean (± SD) abstinence interval was 1.7 (± 1.4) days. Distributions of semen parameters for the study population are presented in Table 1.

**Table 1:** Distributon of semen parameters for study subjects referred for infertility evaluation at Massachusetts General Hospital (n = 306).

parameter	mean	± SD	range
age (yrs)^a^	35.9	5.6	18.2–54.3
volume (ml)	3.0	1.6	0.1–11.0
pH	8.4	0.3	6.8–9.0
concentration (M/ml)	103	95.5	0–655.4
motility (%)	47.6	25.5	0–89.0
morphology (%)^b,c^	6.9	4.7	0–24.0

*Note: *^a^age was not recorded for 5 patients ^b^by Tygerberg-Kruger strict morphology ^c^morphology was not recorded for 2 patients

**Figure 1:**
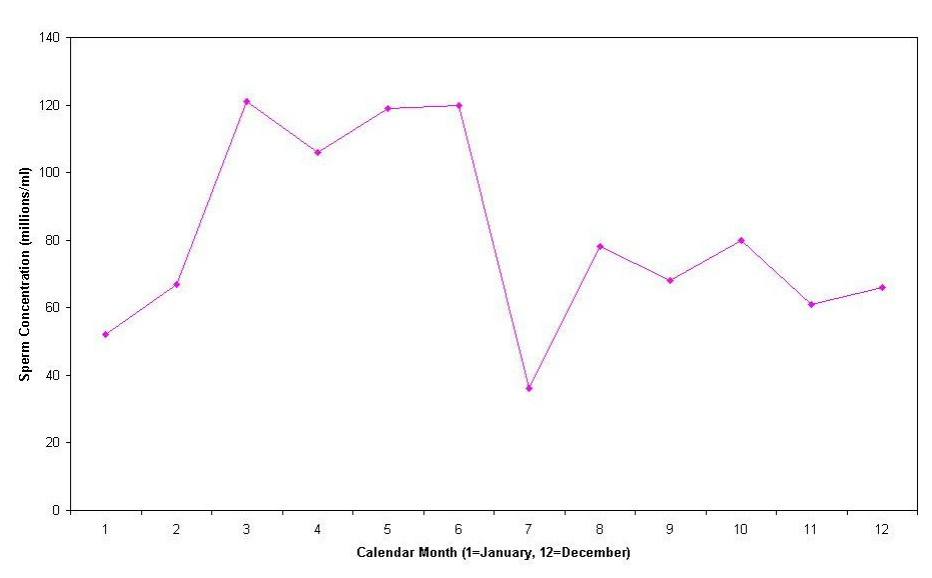
Median sperm concentration by month.

Seasonal variations in semen quality are shown in Table 2. The number of semen samples obtained in the Spring, Summer, Fall, and Winter were 62, 81, 82, 81, respectively. Mean sperm concentration in the spring (137.2 million/ml) was significantly higher than in the winter (99.2 million/ml), summer (93.1 million /ml) and fall (90.6 million/ ml), (p-value < 0.05). These seasonal differences remained after adjusting for age and abstinence times. Figure 1 shows the month-to-month median sperm concentration across the study. Median sperm concentration by month was higher in March, April, May and June than all other months (Figure 1). Mean sperm motility also was higher in the spring (52.3%) than in the summer (47.7%), fall (47.1%) and winter (44.3%). This was higher when compared to winter (*p *= 0.06).

**Table 2:** Seasonal variation in semen parameters observed among study patients referred for infertility evaluation at Massachusetts General Hospital (*n *= 306).

	Spring		Summer		Fall		Winter	
	
Parameter	mean	± SD	mean	± SD	mean	± SD	mean	± SD
volume (ml)	3.0	1.5	2.9	1.6	2.9	1.5	3.1	1.9
PH	8.4	0.2	8.4	0.2	8.4	0.3	8.4	0.3
concentration (M/ml)	137.2^a^	122.1	93.1	80.1	90.6	81.5	99.2	95.3
motility (%)	52.3^b^	22.4	47.7	26.9	47.1	25.1	44.3	26.3
morphology (%)^c^	7.5	4.9	6.7	4.2	7.0	5.1	6.4	4.8

*Note*: Spring = March/April/May (*n *= 62); Summer = June/July/August (*n *= 81); Fall = Sept/Oct/Nov (*n *= 82), and Winter = Dec/Jan/Feb (*n *= 81). ^a ^*p *< 0.05 vs. Summer, Fall, and Winter by multiple regression analysis method ^b^*p *= 0.06 vs. Winter ^c^by Tygerberg-Kruger strict morphology

There were also seasonal variations in sperm morphology parameters. The mean percent normal morphology in Spring (7.5%) was greater than in Summer (6.7%), Fall (7.0%) or Winter (6.4%), though not statistically significant. These seasonal differences remained after adjusting for age and abstinence interval. Mean semen volume and pH were similar across season.

There were no statistically significant relationships between age with semen volume, sperm concentration, percent motility, and percent normal morphology. However, there was little variability in age since the majority of men (> 65%) were between 30 and 40 years old.

In the present study, only 22 men were current smokers and 57 were ex-smokers. After controlling for age and abstinence time, there was no statistically significant relationship between semen quality and smoking status, though current smokers tended to have lower mean sperm concentration (83 million/ml) than never smokers (104 million/ml).

## Discussion

Although there are numerous previously published studies investigating the relationship among semen parameters and season, age and smoking status, the data are not entirely consistent. To determine if these associations are robust, replication is required. It is the accumulation of consistent observations from epidemiological studies that provides confidence in the findings. Therefore, we believe the present study adds to the literature since it provides replication of seasonal trends in semen parameters. In addition, the present study was conducted in men residing in the Northeast region of the United States, an area with distinct seasons and one that has not been well represented in the literature on this topic. Our study has several strengths. The center selected as the site of this study (the Vincent Memorial Andrology Laboratory of Massachusetts General Hospital) has a large and readily accessible population of men seeking infertility evaluation. Based in a large tertiary care facility, the Andrology Laboratory draws patients from diverse backgrounds throughout the New England region of the United States, receiving referrals from physicians in the community and the medical center.

Our study also had potential limitations. Since it is known that there is within-person variability in semen parameters, using a single sample to characterize an individual may introduce measurement error, likely to be random. Another potential limitation is that it is possible that if some men recently moved to the New England area this may introduce bias. However, of the men in the study, 96% of them lived in New England area for at least 3 months prior to their semen analysis, the period of sperm development. Therefore, the concern with recent immigration to the New England area would be minimal.

In the present study, we found higher sperm concentrations, motility and percent normal morphology in the spring than in other seasons. This may partially explain seasonal patterns of births in United States, where there is a deficit of spring births [19], conceived in the summer.

Our data are in agreement with previous reports of seasonal variation in sperm concentration with spring having the highest concentration. Gyllenborg *et al. *[20] found high sperm counts in the spring as compared to the summer. Two other retrospective studies found peak sperm concentrations in the spring and winter [4,21]. In our earlier publication, a retrospective review of semen analysis results from a different cohort of men, we found higher median sperm concentrations in the winter (111.6 million/mL) as compared to the fall (87.7 million/mL), with a median spring concentration of 90.0 million/mL [16]. In our earlier study we were unable to control for abstinence interval, thus if winter abstinence times were longer than spring this may partially account for the differences between studies.

Seasonal variations in sperm morphology were recently explored by Centola and Eberly in a large sample of California men [6]. Although they did not find statistical significant seasonal variations in percent normal morphology, there were significant seasonal differences in percent tail defects, percent tapered forms, and percent immature sperm. In our earlier study we found higher median percentage of sperm with normal morphology in the winter (9.0%) as compared to spring (6.5%) [16]. It is unclear why these results differed from the present study results. The literature on seasonal variations in sperm motility is largely inconsistent [5,6,22,23].

Effects of temperature and hours of daylight may partially explain seasonal variations in semen quality. Sperm production in humans is known to decrease when testicular temperature is raised by experimental techniques [24]. Normal spermatogenesis requires a temperature 2–3°C below rectal temperature [25]. We think temperature and photoperiod may play a role in seasonal variations in semen quality. To explore this further, we are presently conducting a study collecting information on lifestyle factors, such as alcohol and drug use, environmental and occupational exposures, and personal factors such as stress. The information will allow us to further explore risk factors for altered semen quality.

Spermatogenesis occurs over approximately three months and does not seem to vary in duration among men [25]. Chia *et al. *reported there were no significant month-to-month fluctuations in semen volume and sperm density among men who resided in the tropics [26], where there are minimal changes in temperature, unlike the seasonal variation of climate in the New England region. In our study, improved semen parameters in the spring may reflect spermatogenesis during the cold New England winter.

While we did not find a relationship between age and semen parameters, there was little variability in age in our study population. In our earlier study we found inverse associations between age and sperm concentration, motility and morphology [16]. However, the age range was larger in this earlier study. In another study, Schwartz and coworkers found an improvement in semen characteristics up to 25 years of age [27], followed by a leveling off and a subsequent decrease. However, the age relationships were not statistically significant for sperm count, semen volume, and the total number of spermatozoa. Statistically significant decreases in sperm concentration with advanced age were found among 29 'older' fathers (mean age 50.3 years) compared with those from 35 'younger' fathers (mean age 32.2 years) [7]. A recent review of the literature by Kidd *et al. *suggested that increased age was associated with a decline in semen volume, sperm motility, and sperm morphology but not with sperm concentration [8].

Studies have shown associations between cigarette smoking and infertility in women, time to conception, and risk of spontaneous abortion, as well as reduction in fecundity [11,12]. Data on associations between cigarette smoking and male fertility are unclear [11,15,28,29]. Hughes and Brennan [11] reported that there were no consistent effects on semen quality among male smokers. Similarly, a survey of more than 4,000 European couples attempting to become pregnant failed to find an effect of male smoking on fecundity [15].

However, in contrast, in a cross-sectional study of men with proven fertility (n = 243), cigarette smoking was associated with significantly lower semen volume (but not other semen parameters) after adjusting for age and alcohol consumption [29]. Results from a meta-analysis (including >1000 men) indicated that smokers' sperm density is on average 13–17% lower than that of nonsmokers [28]. The available data suggest that cigarette smoking was associated with a significant decrease in sperm density, total sperm count, total motile sperm, and the percentage of normal forms [14].

In our study, only 22 men were current smokers and 57 were ex-smokers. After controlling for age and abstinence time, there was no significant relationship between semen quality and smoking status, though current smokers tended to have lower sperm concentration. The present study is ongoing and we will reexamine these relationships in a larger dataset. In addition, we are also collecting information on other lifestyle and personal factors, such as physical exercise, stress, and alcohol intake. Their relationship with semen parameters will be investigated in the future when we have a larger number of subjects.

## Conclusions

We found seasonal variations in sperm concentration and suggestive evidence of seasonal variation in sperm motility and percent sperm with normal morphology. Although smoking status was not a significant predictor of semen parameters, this may have been due to the small number of current smokers in the study.

## Competeting interests

The authors declare that they have no competing interests.

## Authors' contributions

ZC, LG-B, IS, and RH all contributed equally to this work and reviewed the manuscript.
